# Photocytotoxicity of mTHPC (Temoporfin) Loaded Polymeric Micelles Mediated by Lipase Catalyzed Degradation

**DOI:** 10.1007/s11095-008-9590-7

**Published:** 2008-07-03

**Authors:** Jan-Willem Hofman, Myrra G. Carstens, Femke van Zeeland, Conny Helwig, Frits M. Flesch, Wim E. Hennink, Cornelus F. van Nostrum

**Affiliations:** 1 Department of Pharmaceutics, Faculty of Pharmaceutical Sciences, Utrecht Institute for Pharmaceutical Sciences (UIPS), Utrecht University, P.O. Box 80.082, 3508 TB Utrecht, The Netherlands; 2 Division of Drug Delivery Technology, Leiden/Amsterdam Center for Drug Research (LACDR), Leiden, The Netherlands; 3 Department of Biomedical Analysis, Faculty of Pharmaceutical Sciences, Utrecht Institute for Pharmaceutical Sciences (UIPS), Utrecht University, Utrecht, The Netherlands

**Keywords:** drug release, enzymatic degradation, *meta*-tetra(hydroxyphenyl)chlorin (mTHPC), photodynamic therapy (PDT), polymeric micelles

## Abstract

**Purpose:**

To study the *in vitro* photocytotoxicity and cellular uptake of biodegradable polymeric micelles loaded with the photosensitizer mTHPC, including the effect of lipase-catalyzed micelle degradation.

**Methods:**

Micelles of mPEG750-*b*-oligo(ɛ-caprolactone)_5_ (mPEG750-*b*-OCL_5_) with a hydroxyl (OH), benzoyl (Bz) or naphthoyl (Np) end group were formed and loaded with mTHPC by the film hydration method. The cellular uptake of the loaded micelles, and their photocytotoxicity on human neck squamous carcinoma cells in the absence and presence of lipase were compared with free and liposomal mTHPC (Fospeg®).

**Results:**

Micelles composed of mPEG750-*b*-OCL_5_ with benzoyl and naphtoyl end groups had the highest loading capacity up to 30% (*w*/*w*), likely due to π–π interactions between the aromatic end group and the photosensitizer. MTHPC-loaded benzoylated micelles (0.5 mg/mL polymer) did not display photocytotoxicity or any mTHPC-uptake by the cells, in contrast to free and liposomal mTHPC. After dilution of the micelles below the critical aggregation concentration (CAC), or after micelle degradation by lipase, photocytotoxicity and cellular uptake of mTHPC were restored.

**Conclusion:**

The high loading capacity of the micelles, the high stability of mTHPC-loaded micelles above the CAC, and the lipase-induced release of the photosensitizer makes these micelles very promising carriers for photodynamic therapy *in vivo.*

## INTRODUCTION

Photodynamic therapy (PDT) is an emerging method to treat superficial tumors, for example in the head/neck region, the digestive tract and the skin (1,2). PDT uses a combination of a photosensitizer (PS) and laser light. Upon illumination of the photosensitizer in the presence of oxygen, reactive oxygen species (ROS) are formed. The anti-tumor effect of ROS is exerted either directly by killing tumor cells, or indirectly by damaging tumor-associated vasculature and inducing an immune response against the tumor cells (1–3). PDT has many advantages compared to other cancer treatments, *i.e.* less invasive than surgery, and more targeted than chemotherapy as a result of selective illumination.

**Fig. 1.**
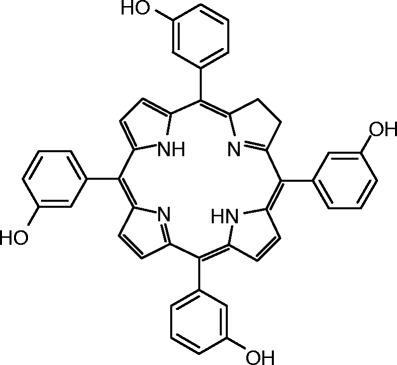
Structural formula of mTHPC (*met*a-tetra(hydroxyphenyl)chlorine, temoporfin).

*Meta*-tetra(hydroxyphenyl)chlorine (mTHPC or temoporfin, Fig. 1) is a second-generation PS, which is currently clinically used as a formulation in ethanol and propylene glycol (Foscan®). It is registered for the palliative treatment of advanced head and neck squamous cell carcinoma (Biolitec AG: Foscan®, summary of product characteristics), and its clinical efficacy has been studied in PDT of other malignancies as well, such as prostate cancer (4), oral squamous cell carcinoma (5), and as adjuvant therapy in combination with surgery in pleural mesothelioma (6). Although mTHPC performs better than other first- and second-generation PSs, like photofrin and *meta-*tetra(hydroxyphenyl)porphyrin (*m*THPP) in terms of potency, depth of PDT effect and daylight induced skin toxicity (1,3,7), further improvement is needed with regard to its formulation and biodistribution. Analogous to other PSs, mTHPC is highly hydrophobic, which complicates its formulation and administration. In patients, precipitation and adsorption of mTHPC to the endothelium of the injected vein and adjacent subcutaneous fat tissue have been observed after intravenous administration of Foscan® (8), and the biodistribution of mTHPC is influenced by its aggregation in the circulation and complexation to serum proteins (9). In addition, skin accumulation obligates patients to prevent exposure to light during the first days after receiving Foscan® (10). The therapeutic index of mTHPC may be greatly improved by enhancing its tumor accumulation and reducing the skin accumulation. To optimize PDT with mTHPC, alternative, water-soluble formulations were developed. PEG–mTHPC conjugates were designed and their efficacy was shown to depend on the linker used to couple PEG to the PS. However, the therapeutic effect of these conjugates was at best similar to native mTHPC (11–13). Encapsulation of mTHPC in bare liposomes (Foslip®) resulted, *in vitro*, in similar cellular uptake and photocytotoxic characteristics compared to Foscan® (8). MTHPC has also been incorporated in PEGylated liposomes (Fospeg®) (14,15). A two to four times higher bioavailability in the tumor, 5.5 times earlier maximal tumor accumulation and four times higher tumor-to-skin ratio compared to Foscan® was found (14), which indicates that PEGylation of the delivery vehicle could be a very interesting approach to formulate mTHPC for *in vivo* use. However, mTHPC is still rapidly redistributed from the Fospeg® formulation to serum components upon intravenous injection (Biolitec AG, personal communication). Although the amount of released mTHPC is not quantified yet, binding of mTHPC to serum components could lead to loss in target specificity. Furthermore, although (PEGylated) liposomes are very promising for *in vivo* application, the release of the contents from these systems can generally not be triggered by environmental factors. Alternatively, polymeric micelles are suitable for the formulation and targeted delivery of PSs to tumors. Their hydrophobic core can accommodate hydrophobic drugs, whereas their hydrophilic shell, which is usually composed of poly(ethylene glycol) (PEG), in combination with their small size (10–100 nm) results in long circulation times, allowing selective accumulation at the tumor site by the so-called enhanced permeability and retention (EPR) effect (16–23). To obtain the best profit of these favorable features, a high loading capacity, *i.e.* drug/polymer ratio, is desired and the drug should be stably incorporated in de micellar core until it reaches the target site. Several PS-loaded micelles have been studied (22), such as Pluronic or PEG2000-distearoylphosphatidyl ethanolamine (DSPE) micelles loaded with meso-tetraphenylporphine (mTPP) (24), aluminium phthalocyanine loaded pH-responsive micelles (25,26), or thermosensitive micelles loaded with solketal-substituted phthalocyanine (27). Micelles that can be triggered by environmental factors to release their contents are of particular interest for selective delivery of PSs to the tumor tissue (25,27–30). Until now there are no formulations available that can stably entrap mTHPC and deliver it directly to the tumor where it is released as a result of an environmental trigger. Therefore, polymeric micelles that degrade at their site of action are attractive systems for the formulation and delivery of mTHPC *in vivo*.

In this study we evaluated the suitability of biodegradable mPEG750-*b*-oligo(ɛ-caprolactone) based micelles for the formulation of mTHPC. These micelles can be easily prepared by the film-hydration method (31), and their small size (sub-20 nm) may result in better properties in terms of biodistribution and tumor penetration, compared to the larger micelles studied so far (32–34). Moreover, it is expected that stable drug incorporation can be obtained, as a result of derivatization of the terminal hydroxyl groups of mPEG-*b*-oligo(ɛ-caprolactone)s with an aromatic group, which may improve the compatibility between the mTHPC and the core, thereby stabilizing the drug-loaded micelles (35–39). Therefore, the loading of mPEG-*b*-oligo(ɛ-caprolactone) based micelles with and without end group modification was studied, followed by examination of the photocytotoxic effect and cellular uptake of the mTHPC loaded micelles, and the influence of lipase, a hydrolytic enzyme, thereon.

## MATERIALS AND METHODS

### Materials

Acetonitrile (ACN), dichloromethane (DCM), tetrahydrofurane (THF), and dimethylformamide (DMF) were obtained from Biosolve Ltd. (Valkenswaard, The Netherlands). MTHPC (*met*a-tetra(hydroxyphenyl)chlorine) and Fospeg® (mTHPC encapsulated in PEGylated liposomes, 1.5 mg/mL mTHPC) were kindly supplied by Biolitec AG (Jena, Germany). Pseudomonas lipase (30 U/mg, 1 U produces 1 μmol glycerol from a triglyceride per min at pH 7.0 at 37°C in the presence of bovine serum albumin) was purchased from Sigma Aldrich Chemie BV (Zwijndrecht, The Netherlands). Phosphate buffered saline (PBS, pH 7.4) was obtained from Braun Melsungen AG (Melsungen, Germany), and was filtered through a 20 nm filter (Anotop®, Whatmann, Breda, The Netherlands) prior to use.

**Fig. 2.**
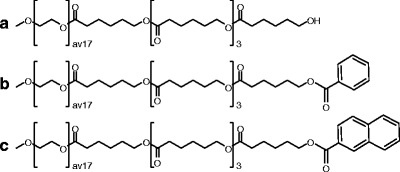
Structural formulas of mPEG750-*b*-oligo(ɛ-caprolactone)_5_ (mPEG750-*b*-OCL_5_-OH) (**a**), benzoylated mPEG750-*b*-OCL_5_ (-Bz) (**b**) and naphthoylated mPEG750-*b*-OCL_5_ (-Np) (**c**). The average degree of polymerization of mPEG750 is 17.

Methoxylated poly(ethylene glycol)-*b*-oligo(ɛ-caprolactone) (mPEG750-*b*-OCL_5_) block copolymers with a monodisperse CL-block and different end groups (Fig. 2) were prepared as described previously (31). In brief, mPEG750-*b*-OCL_5_ was synthesized by ring-opening polymerization of ɛ-caprolactone (20 g, 175 mmol), initiated by mPEG750 (*M*_n_ = 750 Da, 26 g, 35 mmol) and catalyzed by SnOct_2_ (0.71 g, 1.8 mmol) overnight at a temperature of 130°C. To obtain benzoylated and naphthoylated mPEG750-*b*-OCL_5_ (mPEG750-*b*-OCL_5_-Bz, mPEG750-*b*-OCL_5_-Np, respectively) (Fig. 2b and c) the hydroxyl end groups were reacted with a five fold excess of benzoyl- or 2-naphthoyl-chloride in the presence of an equimolar amount of triethyl amine as HCl scavenger. The polydisperse block copolymers were fractionated by preparative reversed phase HPLC (RP-HPLC) to yield monodisperse hydrophobic blocks with exactly five ɛ-caprolactone units, as reported elsewhere (31).

The human head and neck squamous carcinoma cell line UM-SCC-14C (further abbreviated as 14C), originally described by Dr. T.E. Carey (Ann Arbor, MI), was kindly provided by Prof. Dr. G.A.M.S. van Dongen (Department of Otolaryngology/Head and Neck Surgery, VU Medical Center, Amsterdam, The Netherlands). 14C cells were cultured at 37°C in a 5% CO_2_-containing humidified atmosphere in Dulbecco’s modified Eagle’s medium (DMEM) with 3.7 g/L sodium bicarbonate and 4.5 g/L glucose, supplemented with 5% (*v*/*v*) heat-inactivated fetal calf serum, 100 IU/mL penicillin, 100 μg/mL streptomycin sulfate, and 0.25 μg/mL amphotericin B (Gibco, Breda, The Netherlands).

### Formation of mTHPC Loaded mPEG750-*b*-OCL_5_ Micelles

Micelles were formed by the film-hydration method, as described previously (31). Typically, a block copolymer film (10 mg) was formed by solvent evaporation from a 10 mg/mL solution (1 mL) of the polymer in dichloromethane in a 10 mL round bottomed flask. After drying of the film for 30 min under a N_2_ stream, it was hydrated with 1.0 mL PBS at room temperature. The resulting dispersion was filtered through a 200 nm filter (Anotop®, Whatmann, Breda, The Netherlands) (31). Similarly, mTHPC loaded micelles were obtained at different feed ratios by addition of 0.20, 0.30, 0.40, 0.60, or 1.0 mL of a 5.0 mg/mL stock solution of mTHPC in THF to the polymer solution, prior to evaporation of the solvent, representing feed ratios of 10, 15, 20, 30 or 50% (*w*/*w*) mTHPC/polymer, respectively. During all procedures the samples were covered with aluminum foil to avoid photochemical degradation of the photosensitizer.

### Analysis of mTHPC Loaded mPEG750-*b*-OCL_5_ Micelles

The concentration of mTHPC in the micellar dispersions was determined after their dilution in DMF to dissolve the micelles. The absorbance was measured at 417 nm with a Perkin-Elmer Lambda 2 UV–Vis spectrophotometer, using a calibration curve of mTHPC in DMF, which was linear between 0 and 6 μg/mL. The loading efficiency (LE) and loading capacity (LC) were calculated by Eqs. 1 and 2, respectively.

1 $${\text{LE}}\left( \%  \right) = \frac{{{\text{mass}}\;{\text{of}}\;{\text{mTHPC}}\;{\text{loaded}}}}{{{\text{mass}}\;{\text{of}}\;{\text{mTHPC}}\;{\text{fed}}}} \times 100\% $$

2 $${\text{LC}}\left( \%  \right) = \frac{{{\text{mass}}\;{\text{of}}\;{\text{mTHPC}}\;{\text{loaded}}}}{{{\text{mass}}\;{\text{of}}\;{\text{polymer}}\;{\text{added}}}} \times 100\% $$

Absorption spectra were recorded of mTHPC loaded micelles after 20 fold dilution in PBS (final mTHPC concentration 4 μM, polymer concentration 0.5 mg/mL) and of free mTHPC diluted in DMF to the same concentration.

Micelles loaded with 15% (*w*/*w*) mTHPC were analyzed by cryogenic transmission electron microscopy (Cryo-TEM). A Tecnai12 transmission electron microscope (Philips) was used, operating at 120 kV, with the specimen at −180°C and low-dose imaging conditions. Samples were prepared in a temperature and humidity-controlled chamber, using a ‘Vitrobot’. A thin aqueous film of micelle dispersion was formed by blotting a 200 mesh copper grid covered with Quantifoil holey carbon foil (Micro Tools GmbH, Germany) at 25°C and at 100% relative humidity (glow discharged grid; 1 blot during 0.5 s), followed by rapid vitrification by plunging the grid into liquid ethane, and transfer into the microscope chamber using a GATAN 626 cryo-holder system. Images were recorded on a TemCam-0124 camera and processed with AnalySIS software.

The stability of the different samples was visually inspected at 1, 2, 4, and 8 h after preparation, and subsequently every day. Immediately after preparation, a micellar dispersion of mTHPC has a clear, brown-red appearance, whereas turbidity or precipitate formation indicates the presence of free mTHPC, which is hardly soluble in buffer.

### Photocytotoxicity of mTHPC Loaded Micelles

The *in vitro* photocytotoxic effect of mTHPC loaded mPEG750-*b*-OCL_5_-Bz micelles, on 14C carcinoma cells was determined and compared to free mTHPC and liposomal mTHPC (Fospeg®). Two series of mTHPC loaded micelles were prepared. In the first series, a constant polymer concentration was used (10 mg/mL) and the loading with mTHPC was varied from 0.007% to 0.27%. These formulations were 20 times diluted in culture medium, yielding a concentration of mTHPC ranging from 0.05 to 2 μM and a final polymer concentration of 0.5 mg/mL (which is well above the critical aggregation concentration). The second series were prepared by diluting a stock of 15% (*w*/*w*) loaded micelles (1.5 mg/mL mTHPC, 10 mg/mL polymer) in culture medium to the desired concentration range for the *in vitro* PDT experiments, *i.e.* 0.05 to 2 μM mTHPC (final polymer concentration 0.24 to 9 μg/mL). Fospeg® was diluted first in PBS, followed by 20 times dilution in culture medium; free mTHPC was diluted in THF, followed by 200 times dilution in culture medium (final mTHPC concentrations ranging from 0.05 to 2 μM; the final THF concentration of 0.5% (*v*/*v*) has been shown not to affect the cell viability (27)).

14C cells were seeded in a 96-well plate at a density of 2 × 10^4^ cells per well and cultured overnight. Then the medium was removed and 100 μL fresh medium containing the different mTHPC formulations was added. After incubation of the plates for 6 h in the dark at 37°C in a 5% CO_2_-containing humidified atmosphere, the medium was removed, the cells were washed twice with 100 μL PBS, and 100 μL of fresh medium was added.

The plates were illuminated for 10 min with 3.5 mW/cm^2^ light intensity, using a home made device consisting of 96 LED lamps (670 ± 10 nm, 1 LED per well), connected to a water bath thermostated at 37°C. An Orion Laser power/energy monitor (Ophir Optronics LTD, Jerusalem, Israel) was used to measure the light intensity (27). After illumination the cells were incubated overnight in the dark at 37°C in a 5% CO_2_-containing humidified atmosphere, and the number of viable cells was determined by an XTT colorimetric assay (40). The results are expressed as relative cell viability, which is the percentage of viable cells relative to non-treated cells. As a control, for every plate an identical plate was prepared, which was not illuminated.

In a separate experiment, the effect of lipase on the photocytotoxicity of mTHPC loaded micelles was investigated. The same procedure was followed as described above, but before addition to the cells, a micelle-dispersion in medium, containing 0.5 mg/mL mPEG750-*b*-OCL_5_-Bz and 0.4 μM mTHPC was incubated with 0.3 mU/mL of lipase for 48 h at 37°C. It was previously demonstrated that at this lipase-to-polymer ratio the micelles are fully degraded after approximately 30 h (41). As a control, the micelles were incubated without lipase, and to test the effect of lipase on the cells, medium without micelles was incubated with 0.3 mU/mL lipase as well.

### Cellular Uptake of mTHPC Loaded Micelles

14C cells were seeded in a 24-well plate at a density of 15 × 10^4^ cells per well and cultured overnight. The medium was removed, the cells were washed with 500 μL PBS and 500 μL fresh medium containing 10 μM of mTHPC was added. This was either a 200-fold dilution of mTHPC-solution in THF, or a 20-fold dilution of diluted Fospeg®, or two types of micellar mTHPC-samples in PBS, *i.e.* either diluted micelles at mTHPC/mPEG750-*b*-OCL_5_-Bz ratio of 15% (*w*/*w*), or at a relatively high final mPEG750-*b*-OCL_5_-Bz concentration of 0.5 mg/mL in culture medium. The micelles were either freshly prepared or incubated for 48 h at 37°C in the absence or presence of lipase (0.3 mU/mL).

The cells were incubated with mTHPC for 6 h in the dark at 37°C in a 5% CO_2_-containing humidified atmosphere. Subsequently, the medium was removed and the cells were washed twice with 500 μL PBS. The cells were lysed by addition of 500 μL of lysis buffer (pH 8, 50 mM Tris/HCl, 150 mM NaCl, 1% *v*/*w* Triton X-100) followed by incubation on ice for 20 min. The concentration of mTHPC in cell lysate was determined by fluorescence spectroscopy, using a Horiba Fluorolog fluorimeter at a 90° angle with a *λ*_ex_ of 417 and *λ*_em_ of 653 nm. A calibration curve was prepared in DMF with 10% (*v*/*v*) cell lysate, and was linear from 0.73 to 60 nM mTHPC. The cellular protein concentration in a 20 μL aliquot of the cell lysates was determined using the Micro BCA® protein assay (42) (Pierce, Rockford, USA), following the instructions of the supplier. The cellular uptake of mTHPC is expressed as nanomole mTHPC per milligram cellular protein.

## RESULTS

### Loading of mPEG750-*b*-OCL_5_ Based Micelles with mTHPC

mTHPC loaded micelles were prepared by hydration with PBS of a polymer film of mPEG750-*b*-OCL_5_-OH, -Bz, or -Np (see Fig. 2) containing different amounts of mTHPC. Block polymers with 5 caprolactone units were used, because these polymers are able to form sub 20-nm micelles, and have favorable thermal characteristics (Krafft point <4°C; cloud point >37°C) as reported previously (31).

**Fig. 3.**
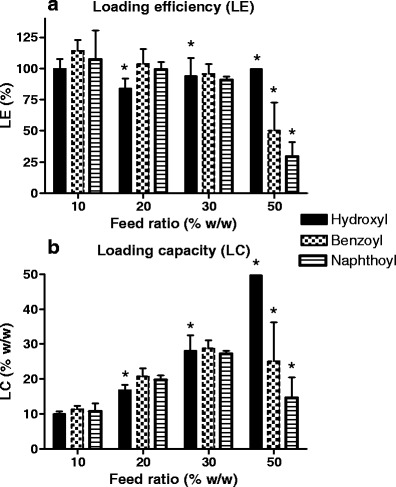
Loading efficiency (**a**) and loading capacity (**b**) of mTHPC into micelles composed of mPEG750-*b*-OCL_5_ with a hydroxyl, benzoyl or naphthoyl end group. The *bars* represent the average ± SD (*n* = 3). The stability of the dispersions marked with an *asterisk* was less than 1 day.

The loading efficiency and loading capacity of the micellar dispersions are presented in Fig. 3a and b, respectively. It was shown that at mTHPC/polymer feed ratios up to 30% (*w*/*w*), the loading efficiency was almost quantitative (80–100%), resulting in loading capacities up to 30% and an mTHPC concentration in the dispersion of 3 mg/mL, which is markedly higher than the aqueous solubility of mTHPC (<100 μg/mL) (Biolitec AG: Physicochemical characteristics of temoporfin). At 10% (*w*/*w*) loading, the dispersions stayed clear for more than a week. At higher loading of 20% and 30% (*w*/*w*), a precipitate was observed in the dispersions of loaded mPEG750-*b*-OCL_5_-OH micelles within 1 day after preparation, indicating that the loading capacity of these micelles was exceeded. In contrast, the micelle dispersions composed of mPEG750-*b*-OCL_5_ with an aromatic end group (Bz or Np) remained clear for more than a week at 20% and 2 days for 30% loading, which demonstrates that these end groups have a favorable effect on the loading and solubilization capacity of the micelles, as shown previously for the taxanes paclitaxel and docetaxel (31,43). At a feed ratio of 50% (*w*/*w*), low loading efficiency (Bz and Np) and/or rapid precipitation in the dispersions (OH) was obtained, which suggests that at this point the loading capacity of all types of micelles was exceeded.

**Fig. 4.**
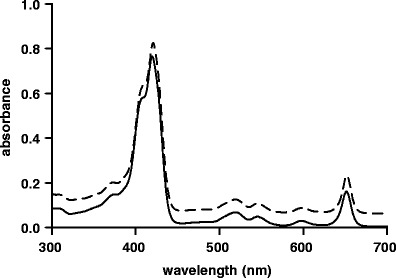
Absorption spectra of mTHPC (4.4 μM) in DMF (*solid line*) and mTHPC-loaded mPEG750-*b*-OCL_5_-Bz micelles (4.4 μM mTHPC, 0.5 mg/mL polymer) in PBS (*dashed line*). The latter spectrum was lifted by 0.05 absorbance units for clarity.

The absorption spectrum of mTHPC formulated in mPEG750-*b*-OCL_5_-Bz micelles in PBS (4.4 μM mTHPC, 0.5 mg/mL polymer) was very similar to that of molecularly dissolved mTHPC (4.4 μM) in DMF (Fig. 4), *i.e.* no broadening or shift of the absorption bands was observed, indicating that mTHPC was molecularly dissolved at this concentration in the micellar core and not incorporated as dimers or larger aggregates (44,45).

**Fig. 5.**
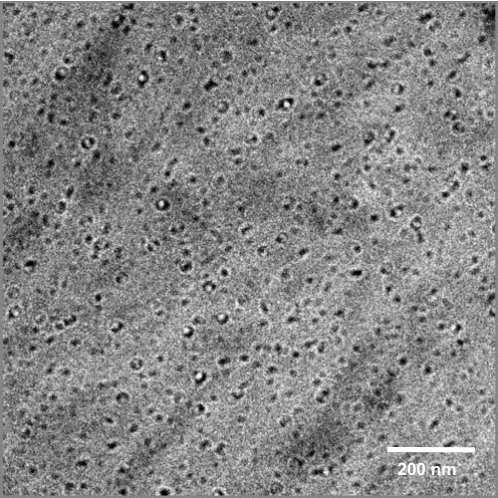
Cryo-TEM image of mPEG750-*b*-OCL_5_-Bz micelles loaded with 15% (*w*/*w*) of mTHPC, at a polymer concentration of 10 mg/mL in PBS. The diameter of the particles is approximately 10 nm.

DLS could not be used to examine the size of the loaded micelles due to absorption of the laser light by the encapsulated mTHPC. Therefore, cryo-TEM was used to visualize the loaded micelles and to determine their size. A cryo-TEM image of mPEG750-*b*-OCL_5_-Bz micelles loaded with 15% (*w*/*w*) mTHPC in PBS (Fig. 5) shows that they have a spherical shape and a diameter of approximately 10 nm. This is in agreement with the results obtained previously with empty or taxane-loaded mPEG750-*b*-OCL_5_-Bz micelles (31,43).

### Photocytotoxicity of mTHPC Loaded Micelles

**Fig. 6.**
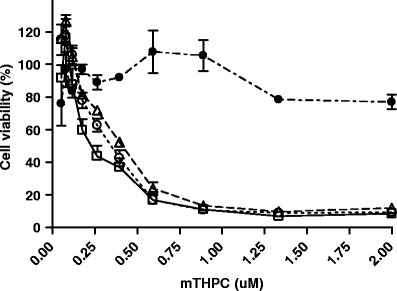
Relative cell viability upon illumination of 14C cells after 6 h of incubation in the presence of free mTHPC (*empty squares*), Fospeg® (*empty triangles*) or mTHPC that was loaded in mPEG750-*b*-OCL_5_-Bz micelles at a fixed mTHPC/polymer ratio of 15% (*w*/*w*) (*empty circles*) or a fixed polymer concentration of 0.5 mg/mL (*filled circles*). Data are presented as average ± SD (*n* = 3). None of the formulations displayed any dark toxicity (results not shown).

The *in vitro* photocytotoxic effect of mTHPC loaded mPEG750-*b*-OCL_5_-Bz micelles on 14C cells was determined after 6 h of incubation and compared with free mTHPC and Fospeg®. Either various dilutions of loaded micelles at a fixed mTHPC/polymer ratio of 15% (*w*/*w*) were used, or the polymer concentration was fixed at 0.5 mg/mL and the mTHPC loading was varied between 0.007% and 0.27% (*w*/*w*) in order to secure a polymer concentration of ≈100 times above its critical aggregation concentration. Figure 6 demonstrates that the effect on the viability of 14C cells of mTHPC-micelles at a fixed mTHPC/polymer ratio is comparable to that of free mTHPC and Fospeg®. The IC_50_ values of the diluted micellar formulation, free mTHPC and Fospeg® were approximately 0.4, 0.2 and 0.3 μM mTHPC, respectively. Remarkably, at a fixed high polymer concentration, the micelles with various loadings were hardly toxic to cells even at the highest mTHPC concentration used, *i.e.* 2 μM, as indicated by a cell viability of 80%.

For the latter formulation the effect of lipase induced degradation of the oligocaprolactone block of mPEG750-OCL_5_-Bz on the photocytotoxicity was studied, at a concentration of 0.4 μM mTHPC (Table I). No dark toxicity was observed for all samples. Interestingly, when the micellar dispersion with 0.5 mg/mL polymer was incubated with lipase prior to adding them to the cells, an extensive decrease in cell viability was observed, *i.e.* approximately 70 times compared to the non-incubated formulation, whereas the lipase did not affect the cell viability, nor did addition of the micelles that were pre-incubated without lipase.

**Table I:** Viability of 14C Cells After 6 h of Incubation with Different Formulations Containing 0.4 μM mTHPC, Relative to Non-treated Cells

Sample	Relative cell viability (%)	
	Dark	After illumination
Micelles, 0.5 mg/mL mPEG750-*b*-OCL_5_-Bz	103 ± 10	87 ± 6
Micelles, 0.5 mg/mL mPEG750-*b*-OCL_5_-Bz, pre-incubated for 48 h at 37°C (no lipase)	97 ± 12	95 ± 6
Micelles, 0.5 mg/mL mPEG750-*b*-OCL_5_-Bz, pre-incubated with 0.3 mU/mL lipase	102 ± 7	1.2 ± 0.7
0.3 mU/mL Lipase (no mTHPC-loaded micelles)	93 ± 10	96 ± 3

Data are presented as average ± SD (*n* = 5–6).

### Cellular Uptake of mTHPC Loaded Micelles

Loaded micelles (with and without pre-incubation with lipase), Fospeg® and free mTHPC were diluted in medium to a concentration of 10 μM mTHPC and incubated with 14C cells for 6 h to determine the cellular uptake of mTHPC (Table II). Fospeg® and 15% (*w*/*w*) mTHPC loaded mPEG750-*b*-OCL_5_-Bz micelles showed a lower cellular uptake of mTHPC compared to free mTHPC, *i.e.* by a factor of 3.6 and 4.8, respectively. More importantly, hardly any mTHPC uptake was measured after incubation with the micelles composed of 0.5 mg/mL mPEG750-*b*-OCL_5_-Bz and 10 μM mTHPC. Interestingly, incubation of the latter formulation with 0.3 mU/mL of lipase resulted in an extensive increase in the uptake of mTHPC by the 14C cells, yielding a cellular uptake comparable to free mTHPC.

**Table II:** Cellular Uptake of mTHPC, Added Either as Free Drug or in Different Formulations, by 14C Cells After 6 h of Incubation

Sample^a^	Cellular uptake (nmol mTHPC/mg protein)
Free mTHPC	1.35 ± 0.33
Fospeg®	0.37 ± 0.11*
15% *w*/*w* mTHPC/mPEG750-*b*-OCL_5_-Bz^b^	0.28 ± 0.09*
Micelles, 0.5 mg/mL mPEG750-*b*-OCL_5_-Bz	0.04 ± 0.02*
Micelles, 0.5 mg/mL mPEG750-*b*-OCL_5_-Bz, pre-incubated for 48 h at 37°C (no lipase)	0.06 ± 0.02*
Micelles, 0.5 mg/mL mPEG750-*b*-OCL_5_-Bz, pre-incubated with 0.3 mU/mL lipase	1.32 ± 0.24

^a^Final mTHPC concentration was 10 μM in each sample. Data are presented as average ± SD (*n* = 6).

^b^Final polymer concentration was 0.045 mg/mL.

*p < 0.05, mean value is significantly different from that of free mTHPC as determined with either the *t* test or the *t* test with Welch correction.

## DISCUSSION

The loading of mTHPC in mPEG750-*b*-OCL_5_ based micelles with three different end groups was studied. Stable mTHPC-loaded mPEG750-*b*-OCL_5_-OH micelles were formed with a loading capacity of 10% (*w*/*w*). This is higher than the loading capacities of other PS-containing micelles, such as meso-tetraphenylporphine (mTPP) in Pluronic micelles (4%) (24), aluminium phthalocyanine in pH responsive micelles (3%) (25), or solketal substituted phthalocyanine in thermosensitive micelles (2%) (27), and comparable to mTPP in PEG2000-DSPE micelles (9%) (24). The excellent loading capacity of mPEG750-*b*-OCL_5_ based micelles may be partly explained by the preparation method of the micelles, as it has been reported that a higher loading of hydrophobic drugs into polymeric micelles can be obtained with the film hydration method when compared to the solvent-evaporation or the dialysis method (36). Interestingly, when block polymers with an aromatic end group were used, even higher loading capacities of up to 28% (*w*/*w*) were obtained, indicating that the loading capacity and stability of these micelles strongly depend on the micellar core structure. In line herewith, it was demonstrated previously that the presence of an aromatic end group favorably affected the encapsulation of taxanes into these micelles (43). The improved loading capacity can be ascribed to a higher hydrophobicity of the OCL_5_-Bz and -Np core as compared to the OCL_5_-OH core (43,46), but likely specific interactions between mTHPC and the micellar core play a role as well. The aromatic mTHPC may form π–π-interactions with the benzoyl or naphthoyl end group, resulting in stable encapsulation in the micellar core. Similarly, improved loading of the aromatic anticancer drug camptothecin into PEG-*b*-poly(β benzyl aspartate)-based micelles when the aromatic content of the core-forming block was increased, was ascribed to this type of interactions as well (36).

The stability of the mTHPC loaded micelles was shown to be an important factor in the *in vitro* experiments. When a stock solution of 15% (*w*/*w*) mTHPC-loaded mPEG750-*b*-OCL_5_-Bz micelles was diluted to obtain a suitable concentration range for *in vitro* PDT, the photocytotoxicity was comparable to that of free mTHPC and similarly diluted Fospeg® (IC_50_ of *ca* 0.3 μM mTHPC in 14C cells, Fig. 6). At these dilutions, the mPEG750-*b*-OCL_5_-Bz concentration (and possibly also the lipid concentration in Fospeg®) is close to or below the critical aggregation concentration (CAC) (31), which means that the micelles are dissociated and mTHPC is actually present in its free form. Interestingly, when a constant high polymer concentration (0.5 mg/mL) well above the CAC of mPEG750-*b*-OCL_5_-Bz (*i.e.* 0.008 mg/mL (31)) was used with varying mTHPC-loadings, no photocytotoxicity was observed up to 2 μM mTHPC, and hardly any cellular uptake of mTHPC, in contrast to free mTHPC and Fospeg® at the same mTHPC concentration (10 μM). After incubation with lipase, which degrades the micelles (41), photocytotoxicity was observed and mTHPC was taken up by 14C cells. This remarkable controlled release property is in striking contrast with the previously observed cytoxicity of paclitaxel and docetaxel formulated in the same mPEG750-*b*-OCL_5_-Bz micelles, which was not dependent on micellar degradation (43).

The reduced uptake for the Fospeg® and 15% (*w*/*w*) mTHPC-loaded mPEG750-*b*-OCL_5_-Bz micelles compared to free mTHPC by a factor 3.6 and 4.8, respectively, (Table II) does not directly correlate with the similar phototoxicity of these three formulations. However, it must be emphasized that the lipid and polymer concentration of Fospeg® and the micellar formulation, respectively, were at more than 5 times higher in the cellular uptake experiments than in the phototoxicity study, due to the detection limits of the uptake studies, and therefore more close to the CAC thereby better retaining the mTHPC inside the particles.

**Fig. 7.**
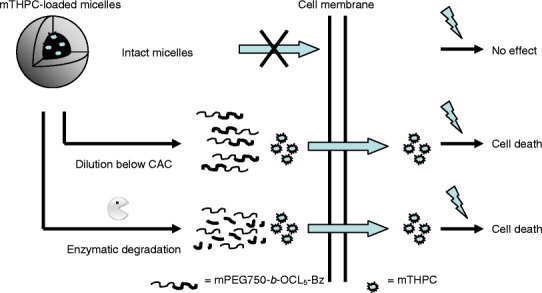
Schematic representation of the processes leading to the photocytotoxicity of mTHPC-loaded mPEG750-*b*-OCL_5_-Bz micelles. Intact mTHPC-loaded micelles are not taken up by the cell, and do not cause cytotoxicity upon illumination. When the loaded micelles are diluted below the critical aggregation concentration (CAC) or enzymatically degraded, mTHPC is released, taken up by the cell, and cytotoxicity is caused upon illumination.

The results of the photocytotoxicity and cellular uptake of the different formulations suggest that when the concentration of polymer is kept well above the CAC, mTHPC remains stably encapsulated in the micelles even in the presence of serum, and that the intact micelles are not taken up by the cells, which may be ascribed to the presence of a dense PEG-layer. Only after dilution of the mTHPC-loaded mPEG750-*b*-OCL_5_-Bz micelles to polymer concentrations below the CAC or after micelle degradation by lipase, the released mTHPC can be taken up by 14C cells and exert its cytotoxic effect upon illumination, as schematically shown in Fig. 7. The relationship between the photocytotoxicity of mTHPC and the integrity of the micelles is clearly different from that observed previously for a solketal-substituted phthalocyanine formulated in thermosensitive micelles (27). At the concentrations used for the photocytotoxicity experiments, the solketal-substituted phthalocyanine was not stably entrapped in de micelles and the observed photodynamic efficacy could be ascribed to diffusion of the photosensitizer out of the micelles. It is anticipated that mPEG750-*b*-OCL_5_-Bz micelles may act as a stable carrier system for mTHPC in the blood circulation because of its stability in serum and inhibited cellular uptake, allowing their selective accumulation at the tumor site after intravenous administration by the EPR effect (20,21). The enhanced lipase activity in tumors compared to healthy tissue (47,48) may cause site-specific micelle degradation and consequent mTHPC deposition.

## CONCLUSIONS

MPEG750-*b*-OCL_5_-based micelles, particularly those composed of polymers with a -Bz or -Np end group have shown interesting features for the formulation of mTHPC. Their high loading capacity and the high stability of mTHPC loaded micelles makes them very promising carriers for PDT with mTHPC *in vivo*. It is expected that accumulation at tumor sites after intravenous administration is feasible, followed by site-specific micelle degradation and subsequent mTHPC release, which will be subject of future studies.
